# Life-Threatening Hemorrhage During Patent Ductus Arteriosus Ligation in a Cat: Xenotransfusion With Canine Blood

**DOI:** 10.3389/fvets.2020.00133

**Published:** 2020-03-10

**Authors:** Julien Dupont, Didier Serteyn, Charlotte Sandersen

**Affiliations:** Department of Clinical Sciences, Anesthesiology and Equine Surgery, Faculty of Veterinary Medicine, University of Liege, Liège, Belgium

**Keywords:** cat, patent ductus arteriosus, hemorrhage, xenotransfusion, canine blood, naturally occurring antibodies

## Abstract

A 13-month-old Sphynx cat was referred for patent ductus arteriosus (PDA) ligation. A left thoracotomy was performed and the PDA was efficiently ligated. Immediately after chest tube placement, it presented extensive intrathoracic bleeding from the caudal intercostal artery. In view of the absolute necessity of a blood transfusion and given that compatible feline blood was not available, xenotransfusion of canine blood was administered to the cat and resulted in a positive outcome.

## Background

Based on a limited number of cases reported in the 1960s, cats do not appear to have naturally occurring antibodies against dog erythrocyte antigens, thereby potentially allowing xenotransfusion of canine blood to cats without inducing severe acute hemolytic reaction (1–5). In view of the limited availability of feline blood and the blood compatibility issues in cats, xenotransfusion has been successfully attempted as a life-saving procedure in a few cases (6–9). Nevertheless, recent advances suggest that cats may actually have naturally occurring antibodies against dog erythrocyte antigens, and acute hemolytic reactions have been reported (10, 11). Regardless of these last advances in interspecies blood compatibility, the case reported here suggests that xenotransfusion of canine blood to cats is an option when compatible feline blood is not available.

## Case Presentation

A 13-month-old, male, Sphynx cat weighing 3.0 kg was referred to the Small Animal Clinic of the Faculty of Veterinary Medicine of the University of Liege for investigation of heart murmur, cough, dyspnea and weakness. Cardiac ultrasonography, performed by a board-certified cardiologist, revealed a left-to-right PDA associated with an enlargement of the left atrium (left atrium to aorta ratio of 2.3), the left ventricle and the pulmonary trunk. Moreover, turbulent blood flow at the aortic valve, increased peak systolic velocity of forward blood flow across the aortic valve (2.7 m/s) and smoke-like echo in the left atrium were observed. Thoracic radiographs confirmed the severe cardiomegaly (vertebral heart size of 11.4) and the dilation of the pulmonary trunk. Furthermore, radiographs demonstrated a dilation of the aortic arch, an increased pulmonary vascularity and were suggestive of a pulmonary edema. Thoracic computed tomography angiography showed a short and large PDA and supported the severe cardiomegaly, the dilation of the pulmonary trunk as well as the increased pulmonary vascularity. All these findings were compatible with a left-to-right PDA associated with a severe cardiomegaly and a left-sided congestive heart failure. The cat was discharged immediately after examination and prescribed furosemide (Furosoral; Dechra) 5 mg PO q12h, clopidogrel (Plavix; Sanofi) 19 mg PO q24h and pimobendan (Vetmedin; Boehringer Ingelhein) 0.9 mg PO q12h; and the surgery was scheduled for 10 days later. In the meantime, dyspnea and weakness improved but coughing frequency increased.

Food and water were withheld for 12 h prior to surgery. A 20 G intravenous catheter was placed in the left cephalic vein and lactated Ringer's solution (Vetivex; Dechra) was infused at a rate of 2 ml/kg/h to replace oral fluid intake. Furosemide, clopidogrel, and pimobendan were administered the evening before surgery. Although the combination of IV fluid therapy and furosemide might seem counterintuitive, it aimed at maintaining normal hydration status without running the risk of worsening congestive heart failure. Preanesthetic physical examination revealed tachycardia [196 beats per minute (bpm)], tachypnea [48 respirations per minute (rpm)], a grade 5/6 heart murmur, a bounding pulse, a strongly positive laryngeal reflex and a body condition score of 4/9; the rest of the examination was unremarkable and the patient was assigned a grade IV according to the ASA health status classification system. Increasing coughing frequency, strongly positive laryngeal reflex and tachypnea were highly suggestive of cardiomegaly and left-sided congestive heart failure. Laboratory analyses were not performed prior to anesthesia.

Methadone (Comfortan; Dechra) (0.2 mg/kg IV) was administered as preanesthetic medication. Anesthesia was induced with midazolam (Midazolam; Mylan) (0.2 mg/kg IV) and alfaxalone (Alfaxan; Dechra) (2 mg/kg IV) and the trachea was intubated with a 3 mm cuffed endotracheal tube. The cat was positioned in right lateral recumbency and connected to a rebreathing circuit. Isoflurane (IsoFlo Vet; Abbott) was delivered in 100% oxygen and pressure-controlled ventilation (Neptune; Medec) was used during the whole procedure to maintain end-tidal carbon dioxide partial pressure below 60 mmHg (respiratory rate: 20–30 rpm, peak inspiratory pressure: 7–12 mmHg, end-expiratory pressure: 0–2 mmHg, tidal volume: 25–40 ml). Mild hypercapnia was tolerated to meet surgical needs. Indeed, increasing minute ventilation, by increasing either respiratory rate or tidal volume, would have prevented surgical access to the PDA. Non-invasive arterial blood pressure was monitored every 5 min using Doppler ultrasonic method (811-B Ultrasonic Doppler Flow Detector; Parks Medical Electronics). Pulse oximetry, electrocardiogram, tidal volume, inspired and expired percentages of oxygen and isoflurane, inspired and expired carbon dioxide partial pressures and esophageal temperature were continuously recorded using a multiparameter monitor (Compact 5S; Datex-Ohmeda).

The cat received alfaxalone (1 mg/kg) to increase the anesthetic depth before the surgery started. Fentanyl (Fentanyl-Janssen; Janssen-Cilag) (3 μg/kg IV) was administered prior to skin incision and infused thereafter throughout the whole procedure (5 μg/kg/h). End-tidal percentage of isoflurane was maintained between 0.7 and 1.4%. A left thoracotomy via the fourth intercostal space was performed and the PDA was ligated within 120 min after induction. Cefazoline (Cefazoline; Sandoz) (20 mg/kg IV) was administered every 90 min until the procedure was completed. Heart rate oscillated between 140 and 180 bpm, systolic arterial blood pressure ranged between 75 and 90 mmHg, peripheral oxygen saturation varied between 99 and 100% and lactated Ringer's solution was infused at a rate of 5 ml/kg/h until the PDA was ligated.

A 14 G guidewire chest tube (guidewire inserted chest tube; Mila) was then inserted using the Seldinger technique. The tube was introduced through a skin incision in the 10th intercostal space and advanced subcutaneously until it reached the 7th intercostal space where it entered the thorax. Immediately after placement of the tube, severe intrathoracic bleeding was observed and the tube was removed. Because surgeons were unable to identify the origin of the hemorrhage, a second thoracotomy was performed via the 7th intercostal space. It allowed identifying the caudal intercostal artery as responsible site for the bleeding. This vessel was thermo-cauterized, which efficiently terminated the hemorrhage. Meanwhile, systolic arterial blood pressure dropped to 40 mmHg, heart rate increased up to 230 bpm and pulse oximeter stopped giving a signal of peripheral oxygen saturation. Two boluses of tetrastarch colloid (Voluven 6%; Fresenius Kabi) (2 ml/kg) and one bolus of lactated Ringer's solution (10 ml/kg) were administered while patient's blood was typed (QuickVet/RapidVet Feline Blood Typing; Zoetis). Systolic arterial blood pressure reached 100 mmHg but the improvement lasted for only 10 min. Blood type was A but there was no feline blood available and it was decided to give untyped canine fresh whole blood that was purposely collected, and given immediately after collection, without performing neither minor nor major cross-match tests. Lactated Ringer's solution infusion was discontinued and transfusion rate started at 5 ml/kg/h and was doubled after 20 min. During transfusion, body temperature, mucous membrane color, capillary refill time, peripheral oxygen saturation, pulse quality, heart rate, heart rhythm, further decrease in arterial blood pressure and possible vomiting, diarrhea, hives, erythema, facial swelling and muscle tremors were closely monitored. At the end of surgery, vacuum was restored in the chest with a 3.5 Fr small animal urinary catheter (Tomcat; Mila) and levobupivacaine (Chirocaine; Abbvie) (1 mg/kg) was instilled through this catheter, which was removed immediately thereafter. Methadone (0.2 mg/kg IV) was administered and the cat recovered in the intensive care unit. The esophageal temperature was 37.5°C after the total anesthesia time of 270 min. The cat became cyanotic after the trachea was extubated, which was efficiently resolved with flow-by oxygen administration. The total amount of blood loss was estimated at 131 ml by weighing soaked compresses and estimating aspirated blood. The cat received 24 ml of blood during surgery and the transfusion was maintained postoperatively until he received the 131 ml in order to match the estimated amount of blood loss. Although the cat remained hemodynamically unstable while receiving blood under general anesthesia (systolic arterial blood pressure as low as 40 mmHg, heart rate of up to 230 bpm and loss of peripheral oxygen saturation signal), it gradually improved and normalized its parameters after transfusion of the whole blood volume. Furthermore, it never showed any sign of fluid overload. Methadone (0.2 mg/kg IV q4 h) was first administered but the analgesic plan was modified and an infusion of fentanyl (2 μg/kg/h) was started 12 h after surgery. Fentanyl infusion was maintained until the third day after surgery where the analgesic protocol was continued with buprenorphine (Vetergesic; Ecuphar) (0.02 mg/kg IV q8h). Furosemide (5 mg PO q12h), clopidogrel (19 mg PO q24h) and pimobendan (0.9 mg PO q12h) were maintained after surgery.

The cat was discharged 3 days after surgery at the owner's request. At that time, it did not show any abnormal clinical sign nor any sign of hemolysis. Hematology revealed a packed cell volume (PCV) of 36% and an absolute reticulocyte count of 142, 000/μL.

The cat was presented for an echocardiographic follow-up at 1 and 2 months after surgery. Although it had developed a hypertrophic cardiomyopathy at that time, it has improved clinically and never showed signs of hemolysis or anemia.

## Discussion

To the best of the authors' knowledge, this is the first reported case of successful xenotransfusion of canine blood to a cat to manage life-threatening hemorrhage after chest tube placement during PDA ligation surgery.

### Prevalence of PDA in the Feline Population

Congenital heart disease has a prevalence of 0.2% in the general feline population and accounts for 5 to 15% of cardiac diseases in cats (12–14). PDA is encountered in 3 to 11.3% of cats with congenital heart disease (12–14).

### PDA Closure Procedures and Associated Complications

Definitive therapy consists in closing the PDA, either by surgical ligation or by transcatheter embolization (15, 16). Although surgical ligation is associated with a higher rate of major intraoperative complication, both procedures have comparable rate of survival to hospital discharge in dogs (17). Perioperative or intraoperative complication rate of surgical ligation ranges from 15.1 to 26.7% in cats (18, 19). Intraoperative hemorrhage is the most common complication and occurs in 6.1 to 13.3% of feline patients (18, 19). Hemorrhage usually results from a tear of the PDA (17, 20). Complications related to chest tube placement are uncommon and include leakage of fluid, subcutaneous emphysema and inadvertent lung laceration (17, 21).

### Blood Transfusion Issues in the Feline Patient

#### Allotransfusion

Feline blood groups are described by the AB system and three blood types are possible: A, B, or AB. Type A and B, but not AB, cats have naturally occurring alloantibodies. Type A cats have weak anti-B alloantibodies and transfused type B erythrocytes only survive for several days. Type B cats have strong anti-A alloantibodies that cause severe acute hemolysis when type A erythrocytes are transfused, erythrocytes only surviving for a few hours (22). Due to the presence of naturally occurring alloantibodies, there is no universal feline donor, while type AB cats are considered as universal recipients and could theoretically receive any blood type (23). Beyond the AB system, other antigens have to be taken into account for transfusion in cats. Indeed, Mik antigens and naturally occurring anti-Mik alloantibodies responsible for hemolytic transfusion reaction have been observed (24). Both typing and cross-matching should therefore be performed prior to any transfusion in cats (25).

Either whole blood (WB) or packed red blood cells (PRBC) can be used to increase blood oxygen-carrying capacity but PRBC are preferred over WB for normovolemic patients since the lack of plasma proteins reduces the risk of transfusion-associated circulatory overload. Volumes to be transfused can be estimated based on the recipient's PCV (Equation 1). When recipient's PCV is unknown, 10–22 ml/kg and 6–10ml/kg are recommended for allotransfusion of WB and PRBC, respectively (25, 26). For both WB and PRBC, it is advisable to start the transfusion at a very slow rate of 0.25–0.5 ml/kg/h for the first 30 min and to increase it up to 10–20 ml/kg/h thereafter (27). However, administration of WB at rates of up to 60 ml/kg/h have been described in cats with hypovolemic shock (28).

$$\begin{array}{l} WB\;\left(ml\right)=Weight\;\left(kg\right)*Blood\;volume\;\left(\frac{ml}{kg}\right)*\\ \;[\frac{PCVrequired\;-\;PCVrecipient}{PCVdonor}] \end{array}$$

(1) $$\begin{array}{r} PRBC\;\left(ml\right)=Weight\;\left(kg\right)*1.5\;ml*Desired\;\%\;increase\;in\;PCV \end{array}$$

Calculation of estimated amounts of allogenic WB and PRBC to be transfused based on the recipient's PCV (27). Estimated blood volume in cats is 50–60 ml/kg and PCV of PRBC is considered around 60%.

The 131 ml of WB transfused in this case corresponded to 44 ml/kg, which was two times higher than the maximum volume recommended for WB allotransfusion. Risk of bleeding should have been more seriously anticipated and PCV measured preoperatively. Moreover, PCV should have been followed before and during transfusion in order to estimate more accurately the blood volume to be transfused and to monitor more closely the response to transfusion. Although the cat was hypovolemic, PRBC might have been used to limit the volume of WB and therefore the risk of transfusion-related circulatory overload. Nevertheless, feline PRBC are not available in our institution. Furthermore, WB offers the benefit of providing clotting factors and platelets, which were surely needed to control such a severe bleeding. Transfusion rate started at 5 ml/kg/h, which was ten times higher than the maximum initial infusion rate recommended for WB transfusion.

Given that hemorrhage is a predictable major complication of PDA ligation and knowing all the blood compatibility issues in cats, typed and cross-matched compatible feline blood should have been available before starting surgery. Although feline blood is often less available and more difficult to collect due to cat size and behavior, the lack of immediately available compatible blood constituted a failure of planning this elective surgery properly.

#### Autotransfusion

Autotransfusion consists in administering autologous blood to a patient, which means blood that has previously been collected from this patient himself. It offers the advantage of reducing the risk of transmission of infectious diseases and of transfusion reactions (27). Three strategies of autotransfusion are currently used:

##### Preoperative autologous blood donation

Preoperative autologous blood donation consists in collecting blood over the weeks prior to a surgical procedure that could potentially be associated with significant blood loss in order to have autologous blood available if hemorrhage occurs (27).

##### Acute normovolemic hemodilution

Acute normovolemic hemodilution consists in collecting blood prior to a surgical procedure that could potentially be associated with significant blood loss but it is performed shortly before or after induction of anesthesia, and the circulating blood volume is restored with crystalloids or colloids. The main benefit is the reduction of red blood cells loss when WB with lower PCV is lost after acute normovolemic hemodilution (27).

##### Cell salvage

Cell salvage consists in collecting and washing blood lost intra- or postoperatively before administering it back to the patient. This technique requires a cell separator system that is not available in our institution (27).

These three strategies have been proven to be efficient in reducing allotransfusion in humans (29–31). Both preoperative autologous blood donation and acute normovolemic hemodilution might have been implemented to the presurgical management of the elective procedure presented here to ward off blood compatibility and availability issues in cats.

#### Xenotransfusion

Several studies have reported successful outcome when canine blood was transfused to cats (1–9). No severe acute hemolysis or other adverse reactions were reported, leading to the assumption that cats were lacking strong naturally occurring antibodies against dog erythrocyte antigens (1–9). However, recipient cats developed antibodies within 4 to 7 days after the first transfusion, leading to a delayed hemolytic reaction (1, 3, 4). Indeed, repeated dog blood transfusion later than 4 to 6 days after the first transfusion led to anaphylaxis with dead frequently ensuing (1, 3, 4). The average lifespan of transfused erythrocytes was <4 days with canine blood while it reached 30 days with compatible feline blood (2, 32).

Even though several studies did not report severe acute adverse reaction when cats were transfused with canine blood, non-fatal acute hemolytic reactions were recently described (1–10). The rapid hemolysis following xenotransfusion suggested that naturally occurring antibodies against canine erythrocytes antigens were present in cats, the prevalence of these antibodies being higher in type A than in type B cats. Non-fatal acute hemolytic reactions occur in cats receiving canine blood, regardless of the canine donor blood type (10, 11). As transfused canine erythrocytes are short-lived, their beneficial effects tend to be very transient (10).

Major and minor cross-match tests should always be performed prior to dog-to-cat transfusion. However, a completely compatible canine blood might be extremely difficult to find. Indeed, a recent study showed that 98.1% of the minor cross-match tests performed between feline recipient's erythrocytes and canine donor's plasma were positive (11). Because canine antibodies are significantly diluted in the feline recipient's blood stream when a small volume of blood is transfused, the clinical significance of positive minor cross-match tests might be limited. Therefore, dogs compatible for major cross-match tests would be preferred over those compatible for minor cross-match tests (11). Nevertheless, as the volume of canine blood transfused in the present case was large, this assumption might not be true and the transfusion might have led to significant hemolysis and worsened the pre-existing anemia.

The cat did not show any sign of acute hemolytic reaction despite it was typed A and neither major nor minor cross-match tests were performed prior to transfusion. Because cats develop antibodies within 4 to 7 days of transfusion, it was recommended to the owner that the patient remained hospitalized for at least 7 days to monitor for possible adverse reactions. When it was discharged, the hematocrit was within normal limits and its absolute reticulocyte count showed regeneration (33).

Xenotransfusion may have a place when compatible feline blood is not available in emergency settings. However, the management of the extensive bleeding that occurred in the case presented here should have been better anticipated as hemorrhage is the most common complication associated with PDA ligation. Extra efforts should have been made to obtain typed and cross-matched compatible feline blood prior to surgery. Otherwise, autotransfusion using either preoperative autologous blood donation or acute normovolemic hemodilution should have been considered.

#### Providing Local Analgesia for Lateral Thoracotomy

Intercostal nerve blocks should have been used as part of the anesthetic protocol. Several studies have proven their beneficial effects for dogs undergoing a variety of thoracic surgeries (34–37). Furthermore, these blocks are fairly easy to perform and do not impose significant risk when performed correctly (38). Adding intercostal nerve blocks would potentially have improved the post-operative comfort of the cat, thereby preventing changes of the analgesic plan.

## Concluding Remarks

PDA is a rare feline cardiac disease that can be efficiently corrected by surgery. Hemorrhage is the most common intraoperative complication associated with PDA closure procedures. Knowing blood compatibility and availability issues in cats, typed and cross-matched compatible feline blood should be available when performing an elective surgery associated with a high risk of bleeding. Nevertheless, when compatible allogenic blood is not available, autotransfusion is a sustainable option that should be considered prior to an elective surgery in cats. Xenotransfusion with canine blood should be regarded as a life-saving alternative in emergency settings but should not compensate for failure in the presurgical planning of an elective procedure.

## Data Availability Statement

The raw data supporting the conclusions of this article will be made available by the authors, without undue reservation, to any qualified researcher.

## Ethics Statement

Ethical review and approval was not required for the animal study because this case report has been written using data collected in a clinical setting. No research has been performed on the patient. While signing the hospitalization contract, the owner is aware and accepts that data concerning his animal might be used for scientific purposes without further consent, guaranteeing confidentiality and anonimity of the owner. Written informed consent was obtained from the owners for the participation of their animals in this study.

## Author Contributions

JD and CS were involved in clinical management of the case. JD, CS, and DS were involved in data treatment, interpretation, and preparation of the manuscript.

### Conflict of Interest

The authors declare that the research was conducted in the absence of any commercial or financial relationships that could be construed as a potential conflict of interest.
